# Data on the acid black 1 dye adsorbtion from aqueous solutions by low-cost adsorbent- Cerastoderma lamarcki shell collected from the northern coast of Caspian Sea

**DOI:** 10.1016/j.dib.2018.01.107

**Published:** 2018-02-07

**Authors:** Hossein Najafi Saleh, Mohammad Hadi Dehghani, Ramin Nabizadeh, Amir Hossein Mahvi, kamyar yaghmaeian, Faraji Hossein, Mansour Ghaderpoori, Mahmood Yousefi, Aliakbar Mohammadi

**Affiliations:** a Torbat Heydariyeh University of Medical Sciences, Torbat Heydariyeh, Iran; b Department of Environmental Health Engineering, School of Public Health, Tehran University of Medical Sciences, Tehran, Iran; c Department of Environmental Health Engineering, School of Public Health, Hamadan University of Medical Health, Hamadan,Iran.; d Department of Environmental Health Engineering, School of Health and Nutrition, Lorestan University of Medical Sciences, Khorramabad, Iran; e Department of Environmental Health, Neyshabour University of Medical Sciences, Neyshabour, Iran; f Students Research Committee, Neyshabur University of Medical Sciences, Neyshabur, Iran

**Keywords:** Acid black1, Adsorption, Dye, Cerastoderma Lamarcki, Low-cos adsorption

## Abstract

The data presented in this article was related to the research article entitled, “The use of Cerastoderma Lamarcki shell for Acid Black 1 adsorption from aqueous solutions.” The characterization data of Cerastoderma Lamarcki shell was analyzed using various instrumental techniques (X-ray diffraction and SEM). The kinetic and isotherm data of pH, initial AB1 concentration, contact time, and CLS dosage were investigated. The optimum conditions for AB1 adsorption using CLS adsorbent were found to be 2 g of adsorbent, pH 2, and a contact time of 60 min. The adsorption data of CLS fit well with the Langmuir model and pseudo-second order model. Finally, the experimental data showed that CLS is a suitable and low-cost adsorbent for the removal of AB1 from aqueous solutions.

**Table:** 

Subject area	Environmental Engineering
More specific subject area	Adsorption
Type of data	Table, image, figure
How data was acquired	Characteristics of the CLS adsorbent were identified with X-ray diffraction and Field Emission Scanning Electron Microscopy. Adsorption of acid black 1 (AB1) by low-cost adsorbent of CLS was examined using batch studies. The effect of different variables such as solution pH (2–11), initial AB1 concentration (50–250 mg l^-1^), contact time (5–240 min), and CLS dosage (2–20 g l^-1^) was investigated. To describe AB1 adsorption on the CLS adsorbent, four types of kinetic models, pseudo-first-order and pseudo-second-order, Elovich and intraparticle diffusion model, were used. The AB1concentration measurement was performed by an atomic absorption spectroscopy (AAnalyst 200 Perkin-Elmer).
Data format	Raw, analyzed
Experimental factors	Shell samples of Cerastoderma lamarcki were collected from the coast of Caspian Sea in Mazandaran province, Iran. CLS were dried in the oven at 85 °C for 12 h. CLS using hammer mill were crushed into the smaller size and it was sieved to 70– 250 μm.
	Data of CLS were acquired for AB1removal from aqueous solution
Experimental features	CLS for dye adsorption from wastewater
Data source location	Neyshabour, University of Medical Sciences, Neyshabur, Iran
Data accessibility	Data are included in this article.

**Value of the data**

• Biochar from CLS was applied to remove Acid Black 1 from an aqueous solution.
• Data in this article, including isotherm and kinetic parameters, is informative for modeling and predicting the adsorbtion capacity of CLS for Acid Black 1 removal.
• The acquired data is advantageous for coastal areas wanting to scale up and design an adsorption column with Cerastoderma lamarcki shells as the medium for removing AB1 from wastewater.

## 1 Data

**Fig. 1:**
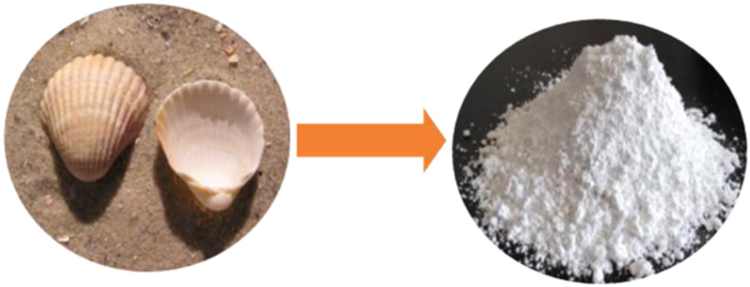
Cerastoderma lamarcki shells and its powder.

**Fig. 2:**
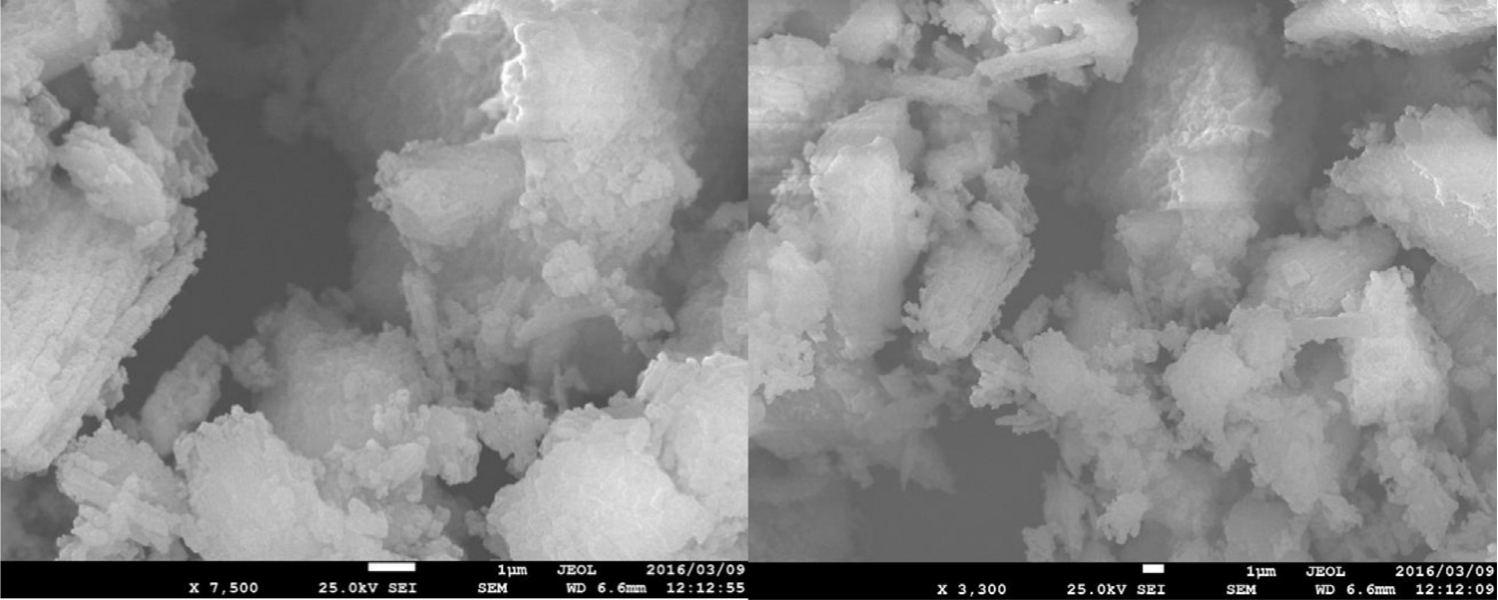
FE-SEM image of low-cost CLS adsorbent.

**Fig. 3:**
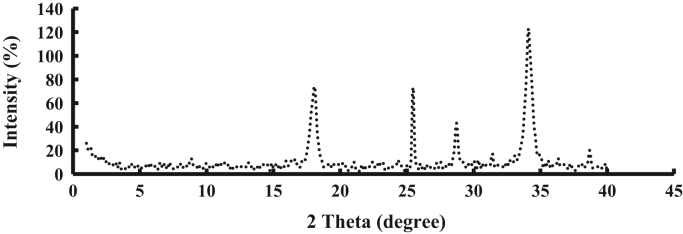
X-ray diffraction spectra of low-cost CLS adsorbent.

**Fig. 4:**
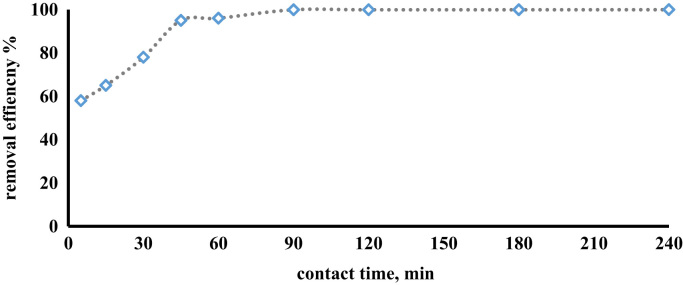
Effect of contact time on AB1 adsorption on CSL adsorbent (pH = 2, adsorbent dosage = 7 g l^-1^).

**Fig. 5:**
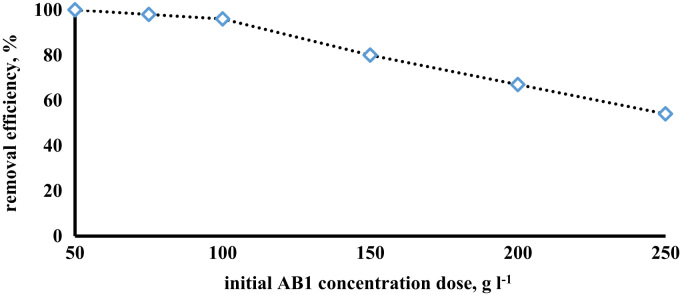
Effect of initial AB1 concentration of on adsorption on CLS adsorbent (pH = 2, adsorbent dosage = 7 g l^-1^, contact time = 60 min).

**Fig. 6:**
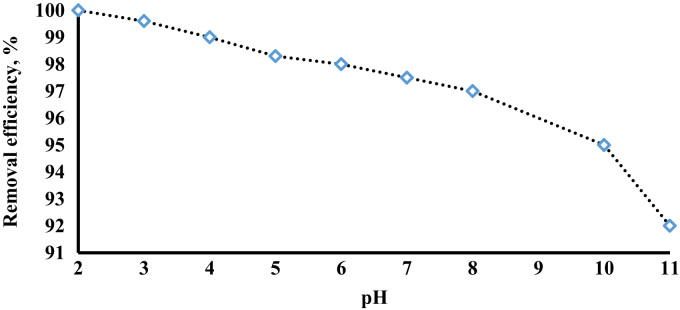
Effect of pH variations on AB1 adsorption onto CLS (adsorbent dose = 7 g l^-1^, contact time = 60 min).

**Fig. 7:**
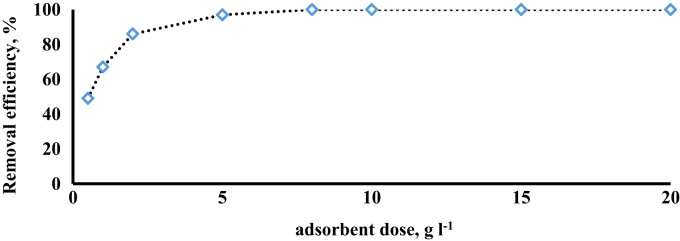
Effect of adsorbent dose on AB1 adsorption onto CLS adsorbent (initial AB1 concentration = 50 g l^-1^, pH = 2, contact time = 60 min).

The prepared CLS adsorbent was in the form of a powder (Fig. 1). The morphology of the CLS adsorbent is shown in Fig. 2. The crystal structure of CLS was studied by x-ray diffraction (Fig. 3). The kinetic, isotherm, and thermodynamic parameters were estimated using models listed in Table 1. Data on the isotherms and kinetics for adsorption of chromium ions onto Cerastoderma lamarcki shell is presented in Table 2, Table 3. Fig. 4, Fig. 5, Fig. 6, Fig. 7 present the comparison data for AB1 adsorption by CLS for the parameters of contact time, initial AB1 concentration, pH, and CLS dosage, respectively.

**Table 1:** Empirical formulas of the applied kinetic models used in this study [3].

**Models type**	**formula**	**plot**
Pseudo first order	$\log(q_{e}-q_{t})=log(q_{e})-\frac{k_{1}}{2.303}t$	log (q_e_-q_t_) vs. t
Pseudo second order	$\frac{1}{q_{t}}=\left(\frac{1}{k_{2}{q_{e}}^{2}}\right)+\left(\frac{1}{q_{e}}\right)t$	t/q_t_ vs. t
Elovich	$q_{e}=\left(\frac{1}{\beta }\right)ln(\alpha \beta )+\left(\frac{1}{\beta }\right)\ln\;t$	In t vs. q_t_
Intra-particle diffusion	$q_{t}=k_{dif}t^{0.5}+C$	q_t_ vs. t^1/2^

**Table 2:** Kinetic constants for AB1 adsorption using CLS adsorbent.

**Isotherm type**	**Isotherm parameters**	**AB1 Concentration (mg l**^**-1**^**)**		
		**50**	**100**	**200**
Psudo first order model	K_1_	0.033	0.046	0.015
	R^2^	0.831	0.819	0.815
	q_cal_	1.619	3.474	2.339
Psudo second order model	K_2_	0.046	0.022	0.038
	R^2^	0.999	0.999	0.999
	q_m_	5.112	10.233	8.953
Elovich	α	1.691	0.293	0.274
	β	1.127	0.563	0.718
	R^2^	0.903	0.904	0.787
Intraparticle diffusion	K_dif_	0.266	0.532	0.397
	R^2^	0.668	0.669	0.526
	C	1.953	3.905	4.349

**Table 3:** Isotherm model constants for AB1 adsorption onto CLS adsorbent.

**Isotherm type**		**Isotherm parameters**	**Value**
Fraundlich		n	2.022
		K_f_	1.473
		R^2^	0.889
Langmuir	I type	K_L_	0.039
		R^2^	0.983
		q_m_	15.877
	II type	K_L_	0.025
		R^2^	0.991
		q_m_	20.894
	III type	K_L_	0.042
		R^2^	0.75
		q_m_	15.6
	IV type	K_L_	0.031
		R^2^	0.75
		q_m_	18.133

## 2 Materials and methods

### 2.1 Materials

Acid black 1 (80% purity), HCl, and NaOH (to adjust pH) were supplied by Sigma-Aldrich. All chemical materials required in this study were purchased from Merck Co. Double-distilled water was used to prepare working solutions.

### 2.2 Preparation of biosorbent

Samples of Cerastoderma lamarcki shell (CLS) were collected from the coast of the Caspian Sea in Mazandaran province, Iran. After collection, the shells were washed with tap water to remove any dirt or other contaminant. After the initial wash, they were washed twice more with deionized water. Then, the shells were dried in an oven at 85 °C for 12 h. Next, they were crushed using a hammer mill and sieved to 70–250 μm. Finally, the end product was stored in a polyethylene container for later use. Fig. 1. shows the Cerastoderma Lamarcki shells [1], [2], [3], [4], [5], [6], [7].

### 2.3 Design of experiments

2.3. Experimental Design.

The adsorption of Acid Black 1 (AB1) by the low-cost adsorbent CLS was examined using batch studies. The effects of different variables, namely solution pH (2–11), initial AB1 concentration (50–250 mg l^-1^), contact time (5–240 min), and CLS dosage (2–20 g l^-1^) were investigated. Initially, the stock solution of AB1 (1000 mg l^-1^) was prepared with double-distilled water and stored under standard conditions [8]. AB1 concentrations were prepared by proper dilution (C_1_V_1_ = C_2_V_2_) using the stock solution. To start the tests, a 250-ml Erlenmeyer flask was employed. Then, certain amounts of the stock solution and CLS were added. To obtain optimum contact time, 25 ml of the stock solution prepared by dilution was poured into the flask; 0.7 gr (7 g l^-1^) of adsorbent was added at an adjusted pH of 3. The samples were placed in a shaker and shaken at a constant rate of 150 rpm for various time periods. Each CLS dosage was added to 100 ml of AB 1 solution. The solution pH was adjusted using 0.1 M HCl and NaOH. After experiments, the remaining adsorbent was separated from the solution by centrifugation (3500 rpm, 10 min). Then, the residual AB1 concentration was determined by spectrophotometry (UV-UVIS, 622 nm). The experiments were conducted at the constant temperature of 25 ± 1 °C [2], [8]. Finally, the amount of AB1 adsorbed onto the CLS adsorbent was calculated using Eq. 1 [8]:

(1) $$q_{e}=\frac{V(C_{o}-C_{e})}{m}$$

Where, C_o_ and C_e_ are the initial and final concentrations of AB1 in solution (mg l^-1^), respectively, V is the volume of AB1 solution (ml), and m is the weight of the CLS (g). The removal efficiency of AB1 was calculated using Eq. 2 [9]:

(2) $$R,\%=\frac{\left(C_{0}-C_{t}\right)}{C_{0}}$$

Where, *C*_o_ and *C*_t_ represent the initial and t AB1 concentrations (mg l^-1^), respectively. All stages were repeated several times to determine optimum pH, CLS dosage, and AB1 concentration values.

### 2.4 Equilibrium adsorption modeling

Isotherm models such as Langmuir and Freundlich were applied to determine the relationship between equilibrium capacity (*q*_e_) and equilibrium concentration (*C*_e_). Adsorption kinetic models were used to predict the rate of adsorption and adsorption mechanisms. To describe AB1 adsorption on the CLS adsorbent, four kinetic models (pseudo-first-order, pseudo-second-order, Elovich, and intraparticle diffusion) were used [9], [10].
