# Supplementary material for: Data on the acid black 1 dye adsorbtion from aqueous solutions by low-cost adsorbent- Cerastoderma lamarcki shell collected from the northern coast of Caspian Sea
Source: Data Brief. 2018 Feb 7;17:774–80. doi: 10.1016/j.dib.2018.01.107 (PMC5988499; doi:10.1016/j.dib.2018.01.107)
Supplement: Supplementary file 1 — Supplementary material [file mmc1.docx]

**Conflict interests form**

**The authors declare no Conflict of Interest.**
